# Effects of Different Dietary Crude Protein Levels on Reproductive Performance, Egg Quality and Serum Biochemical Indices of Wanxi White Geese in the Laying Period

**DOI:** 10.3390/ani15081140

**Published:** 2025-04-15

**Authors:** Wenlong Shi, Changsheng Jiang, Xu Zhang, Qianqian Hu, Chunfang Zhao, Xiaojin Li, Ahmed H. Ghonaim, Shenghe Li, Man Ren

**Affiliations:** 1 Anhui Provincial Key Laboratory of Animal Nutritional Regulation and Health, College of Animal Science, Anhui Science and Technology University, Fengyang 233100, China; 18269749049@163.com (W.S.); jiangcs@ahstu.edu.cn (C.J.); 18010919050@163.com (X.Z.); huqq@ahstu.edu.cn (Q.H.); zhaocf@ahstu.edu.cn (C.Z.); lixj@ahstu.edu.cn (X.L.); 2 Anhui Engineering Technology Research Center of Pork Quality Control and Enhance, College of Animal Science, Anhui Science and Technology University, Chuzhou 233100, China; 3 National Key Laboratory of Agricultural Microbiology, College of Animal Sciences and Veterinary Medicine, Huazhong Agricultural University, Wuhan 430070, China; a.ghonaim@webmail.hzau.edu.cn; 4 Desert Research Center, Cairo 11435, Egypt

**Keywords:** crude protein, Wanxi white geese, reproductive performance, egg quality, serum biochemical indices

## Abstract

Crude protein (CP) in diets is essential for maintaining animal health and production performance. However, the protein requirements of Wanxi white geese during the laying period are not well understood. In this study, 120 one-year-old Wanxi white geese were selected and divided into three groups based on similar body weights, namely 14% CP, 15% CP, and 16% CP, with each group consisting of 40 animals. The feed was administered for 120 days. Compared with the 14% CP group, the 15% CP group showed a significant increase in the number of courtships and matings, a reduction in nesting frequency, an enhancement in the egg fertility, and an improvement in the nutritional components, and specific gravity of eggs. Additionally, the 16% CP group promoted the secretion of serum E2, LH, P4, and GnRH while inhibiting the secretion of LEP, compared with the 14% CP group. Taken together, it can be seen that a diet containing 15% CP can enhance the reproductive performance, egg fertility, and egg quality of Wanxi white geese. This study is the first to analyze the effects of different dietary CP levels on the reproductive performance and egg specific gravity of Wanxi white geese during the laying period, providing a theoretical basis for formulating feeding standards for this breed.

## Simple Summary

This study investigated the effects of dietary crude protein (CP) levels on the reproductive performance and egg quality of Wanxi white geese during their laying period. Three groups of geese were fed diets containing 14%, 15%, or 16% CP for 120 days. The results indicated that the 15% CP group exhibited enhanced courtship behavior, increased mating frequency, higher egg fertility, and improved egg specific gravity compared to the 14% CP group. Additionally, the 16% CP diet was associated with elevated reproductive hormone levels (E2, LH, P4, GnRH) but resulted in reduced leptin (LEP) levels. Overall, a 15% CP diet was identified as optimal for enhancing reproduction and egg quality in Wanxi white geese, providing key data for future feeding standards.

## 1. Introduction

There are many excellent medium-sized goose breeds in China, such as the Wanxi White Goose [1], and Zhejiang goose [2], known for their large size, rapid early growth, strong disease resistance, and good meat quality. They are especially valued for their high quality of down. The Wanxi white goose is recognized as an outstanding medium goose breed in China, characterized by its large size, good meat quality, high quality of down, and strong disease resistance. However, this breed is a seasonal egg-layer. Due to its long-term reliance on natural hatching, the Wanxi white goose experiences a short laying period, a prolonged layoff period, low natural reproductive performance, and strong broodiness, resulting in an average annual egg production of fewer than 30 eggs [3].

The crude protein (CP) content in animal diets varies significantly due to differences in physiological structure and habits among various livestock and poultry [4]. Even slight increases or decreases in dietary crude protein levels can impact animal performance, including production efficiency, growth performance, and variations associated with gender and growth stage. Therefore, adjustments to dietary CP levels should meet the growth, development, and production performance requirements of different poultry. Currently, there are no uniform feeding standards for Chinese native geese, leading farmers to rely on personal experience and chicken feeding standards to determine the nutritional requirements for the different feeding stages of goose development [5]. Geese are herbivorous waterfowl, and their cecum possesses a remarkable ability to decompose and utilize fibrous materials. On this basis, preparing reasonable nutrition plan is a key step toward exploring the efficient and sustainable development in the goose breeding industry [6].

From the 1970s to the early 2020s, research on the crude protein level requirements of geese at different stages has been ongoing. Ashour and colleagues have found that a diet containing 16% crude protein significantly improved the growth performance, carcass traits and meat quality of Egyptian geese aged 12–24 weeks [7]. Another study indicated that 3-year-old Yili geese fed a 15.2% protein diet for 10 weeks during the laying period exhibited increased serum reproductive hormone levels, up-regulated gene expression of sex hormones, down-regulated prolactin expression, and increased egg production rates, while also reducing nesting rates and prolonging laying periods [8]. Furthermore, a study has reported that a diet with 16% crude protein maintained the growth performance and other physiological indices of Egyptian geese aged 1–12 weeks [9]. Research has shown that the body weight and nitrogen utilization rates of Jiangnan white geese aged 1 to 30 days improved significantly with an 18.5% animal protein (fish meal) diet [10]. Xing Guo gray geese fed a diet containing 14.11% protein and 5% crude fiber from 1 to 21 days of age showed enhanced gut microbial balance and the prevention of gout [11]. Other findings have suggested that the optimal dietary protein level for geese is around 18% [12]. In addition, the fecundity of poultry is regulated through various mechanisms, including central nervous regulation, hormonal regulation, and immune system regulation. The coordination and modulation of reproductive hormones such as gonadotropin-releasing hormone (GnRH), follicle-stimulating hormone (FSH), luteinizing hormone (LH), prolactin (PRL), estradiol (E2), and progesterone (P4) maintain the reproductive activities of poultry [13]. Follicle stimulating hormone receptors (FSHRs) play a vital role in the development, maturation, and ovulation of follicles, regulating overall follicular development [14]. FSH and LH levels increase with age, peaking at sexual maturity, while E2 and P4 levels rise after reaching sexual maturity [15]. Excessive dietary protein in poultry can lead to reduced growth performance, increased metabolic burden, and higher nitrogen excretion, contributing to environmental pollution. Overconsumption may also impair kidney function due to elevated urea production and increase feed costs without improving productivity. Therefore, optimal protein levels are crucial for balancing health and economic efficiency. Currently, there is no scientific study characterizing the protein requirements of Wanxi white geese during the laying period.

Therefore, in this study, we designed three dietary formulations with varying crude protein levels to be fed throughout the complete laying period. By analyzing the effects of these varying crude protein levels on reproductive behavior, ovarian microstructure, egg production performance, egg quality, and serum reproductive hormone levels, we aimed to identify the optimal dietary crude protein level for this breed. This research provides a scientific basis for formulating feeding standards for Wanxi white geese during their laying period.

## 2. Materials and Methods

### 2.1. Animal Ethics Statement

All animal experiments were approved by the Animal Ethics Committee of Anhui Science and Technology University, under protocol number AK2023026. All experimental procedures were carried out in strict accordance with the “Guidelines for the Care and Use of Test Animals” of Anhui Province.

### 2.2. Experimental Animals

In this study, 120 one-year-old Wanxi white geese (with a sex ratio of 1:4 and an average weight of 6.03 ± 0.88 kg) were randomly divided into three treatment groups: 14% CP, 15% CP, and 16% CP, with 4 pens per group and 10 geese per pen. The nutrient levels in the feed were determined using chemical analysis methods. The experimental geese were raised in confinement with natural lighting and free access to feed and water, and the temperature was maintained at around 26 °C. The feeding trial lasted for 120 days. The composition and nutritional components of the experimental diet are shown in Table 1. Breeding management was carried out in accordance with the “Technical Specifications for Breeding Goose Breeding” (NY/T 3446-2019) [16], which included the daily cleaning of the exercise area and regular ventilation and disinfection. Daily observations were conducted to record the number of courtships, matings, nesting behavior, and egg production of the Wanxi white geese.

### 2.3. Sample Collection

The laying period of Wanxi white geese lasted for six months. In the third month of the laying period, 10 female geese were randomly selected from each group for blood collection via the sub-wing vein (without euthanasia), and the serum was stored at −80 °C for the determination of biochemical indicators and hormone concentrations. Additionally, 20 goose eggs were randomly collected from each group for egg quality assessment. In the fourth month of the laying period, another 10 female geese per group were randomly selected, euthanized via ether anesthesia, and their ovaries were collected and fixed in 4% paraformaldehyde for ovarian tissue structure observation.

### 2.4. Detection of Serum Biochemical Indices

The concentrations of total cholesterol (TC), total protein (TP), alanine aminotransferase (ALT), alkaline phosphatase (ALP), urea nitrogen (BUN), and uric acid (UA) in serum were determined using kits purchased from Nanjing Jiancheng Biotechnology Co., Ltd. (Nanjing, China). The biochemical indicators were measured according to the manufacturer’s instructions.

The concentrations of estradiol (E2), follicle-stimulating hormone (FSH), gonadotropin releasing hormone (GnRH), leptin (LEP), luteinizing hormone (LH), progesterone (P4), and prolactin (PRL) in serum were measured using kits specifically custom-manufactured by Nanjing Jiancheng Biotechnology Co., Ltd. for geese. The kits used in this study are listed in Table 2.

### 2.5. Tissue Staining

After fixation, the ovarian tissues were embedded in paraffin, and thin sections were then prepared from the paraffin-embedded tissues. These sections were subsequently stained with hematoxylin and eosin (H&E). A five-point sampling method was used for image acquisition, and the number of follicles in each image was counted and graded [17].

### 2.6. Determination of Egg Quality

In the third month of the laying period, 20 eggs were collected from each group (totaling 60 eggs) for egg quality assessment. The albumen height was measured using an albumen height determinator (Bulader-DA100, Bulader, Beijing, China). Briefly, the egg was placed on the glass plate of the analyzer to determine the height of the thick albumen at the midpoint between the edge of the egg yolk and the edge of the thick albumen. Measurements were taken at three equidistant points to obtain the mean value.

Egg gravity analysis was assessed using the saline flotation method, a classical density measurement technique based on Archimedes’ principle. Nine beakers were each filled with 1 kg of water, with 68 g of salt added to the first beaker. The specific gravity value was calibrated using a gravimeter and set as grade 0. Subsequently, an additional 4 g of salt was added to each of the remaining beakers in succession, creating solutions of grades 1–8. The specific gravity values for solutions of grade 1–8 are listed in Table 3. The eggs were gradually placed into the saline solution starting from grade 0 until each egg was suspended, indicating its specific gravity.

The egg yolk ratio was calculated as (egg yolk weight/egg weight) × 100%, while the egg white ratio was calculated as (egg white weight/egg weight) × 100%. The egg shape index was determined as the ratio of the longitudinal diameter to the transverse diameter, with measurements taken using a vernier caliper. Eggshell thickness was measured as the average thickness at the blunt, middle, and sharp ends of the eggshell using a micrometer (211-101F, Guanglu, Guilin, China). The egg yolk color was measured using a colorimeter (CR-10, Konica Minolta, Chiyoda, Japan).

The egg qualification rate is calculated as the number of qualified eggs divided by the total number of eggs. Unqualified eggs include those weighing greater than 250 g or less than 120 g, as well as those with surface pitting, soft shells, deformities, or cracks.

### 2.7. Determination of Nutrients in Eggs

The contents of water, crude fat, crude protein, and amino acids in eggs were measured to evaluate the nutritional composition of goose eggs [18]. The moisture content was determined using the direct drying method [19], a classical analytical technique that measures mass loss after heating to remove volatile components (primarily water). Crude fat was measured by the soxhlet extraction method [20], while crude protein was determined using the Kjeldahl’s nitrogen determination method [21]. The concentrations of amino acids, including aspartate, threonine, serine, glutamic acid, glycine, alanine, valine, methionine, isoleucine, leucine, tyrosine, phenylalanine, lysine, histidine, arginine, and proline were determined according to the GB/T 14965-1994 standard [22].

### 2.8. Statistical Analysis

The obtained results were expressed as the mean ± standard errors of the mean (SEM). Statistical analysis was performed using ANOVA in SPSS 20.0 software (IBM Corp., Armonk, NY, USA). Each pen contained 10 geese, and pen means were used as replicates for analysis. Post hoc comparisons were performed using Duncan’s test when ANOVA indicated significant treatment effects. In the results, different capital letters indicate highly significant differences (*p* < 0.01), different lowercase letters indicate significant differences (*p* < 0.05), and the same letter indicates non-significant differences (*p* > 0.05) within the same line.

## 3. Results

### 3.1. Reproductive Behavior and Performance of Wanxi White Geese

Courtship behavior in Wanxi white geese can be categorized into three main types: male geese courting female geese, female geese courting male geese, and mutual courtship between both sexes. In this study, the courtship behaviors of Wanxi white geese fed diets containing 14% CP, 15% CP, and 16% CP were observed. It was found that the number of courtships by male geese in the 15% CP group was significantly higher than that in the 16% CP group (*p* < 0.05). No significant differences were observed in other courtship methods. Additionally, compared with the 14% CP and the 16% CP groups, the 15% CP group significantly increased the number of instances of male geese climbing over female geese, female geese climbing over male geese, and mating occurrences (*p* < 0.05 or *p* < 0.01). While the 15% CP diet significantly reduced the nesting frequency of female geese compared with the 16% CP group (*p* < 0.05), there was no significant difference when compared with the 14% CP group (*p* > 0.05). Furthermore, the 15% CP diet significantly enhanced the fertilization rate of goose eggs compared with both the 14% CP and 16% CP groups (*p* < 0.01), although it did not affect the hatching rate of goose eggs (*p* > 0.05) (Table 4). These results indicate that a diet containing 15% CP significantly promotes the reproductive behavior and performance of Wanxi white geese during the laying period.

### 3.2. Reproductive Hormones in Wanxi White Geese During the Laying Period

As shown in Table 5, the concentration of ALT in the 16% CP group was significantly lower than that in the 15% CP group (*p* < 0.05). Compared with both the 14% CP and 16% CP groups, the concentration of serum UA in the 15% CP group was significantly decreased (*p* < 0.05), indicating that a 15% CP diet may reduce the risk of gout in Wanxi white geese. However, different CP levels in the diet did not affect the concentration of TC, TP, ALP, BUN, and CA in Wanxi white geese during the laying period (*p* > 0.05).

As demonstrated in Table 6, the concentrations of serum E2, LH, P4, and GnRH in the 14% CP group were significantly higher than those in the 15% CP group (*p* < 0.05 or *p* < 0.01). Furthermore, dietary supplementation with 16% CP significantly increased the concentrations of E2, LH, P4, and GnRH in serum compared with the 15% CP group (*p* < 0.01). The concentration of LEP in the 15% CP group was significantly higher than in both the 14% CP and 16% CP groups (*p* < 0.01). In addition, the concentration of serum LEP in the 14% CP group was significantly higher than that in the 16% CP group (*p* < 0.01). Different CP levels in the diet did not affect the concentrations of PRL and FSH in Wanxi white geese during the laying period (*p* > 0.05). These results indicate that a diet containing 16% CP can increase the concentrations of reproductive hormones of Wanxi white geese during the laying period.

### 3.3. Ovarian Development of Wanxi White Geese During the Laying Period

The histological structure of the ovaries of Wanxi white geese during the laying period was examined, and the number of follicles was recorded. As shown in Table 7, compared with the 16% CP group, the number of small white follicles in the 14% CP group was significantly reduced (*p* < 0.05), while the number in the 15% CP group was significantly lower (*p* < 0.01). In addition, the number of large white follicles in the 16% CP group was significantly higher than that in the 14% CP and 15% CP groups (*p* < 0.05). The number of large yellow follicles in the 15% CP group was also significantly decreased compared with the 16% CP group (*p* < 0.05).

As shown in Table 8, dietary crude protein level had no significant effect on the average egg weight, daily egg production, and feed-to-egg ratio of Wanxi white geese (*p* > 0.05). The qualified rate of eggs in the 14% CP group was 96.0%, with an average production of 18.7 eggs per goose. In the 15% CP group, the qualified rate was 94.8%, with an average of 19.8 eggs produced per goose. The 16% CP group had a qualified rate of 96.3%, with an average production of 20 eggs per goose.

### 3.4. Egg Quality of Wanxi White Geese

As shown in Table 9, the eggs’ specific gravity and yellowness (b) of the 16% CP group were significantly reduced compared with the 15% CP group. However, different CP levels in the diet had no significant effect on the egg shape index, eggshell thickness, albumen height, egg yolk color, egg yolk ratio, egg white ratio, brightness (L), and redness (a).

### 3.5. Nutritional Quality of Wanxi White Goose Eggs

As shown in Table 10, the content of crude fat in the 16% CP group was significantly higher than that in the 14% CP group (*p* < 0.05), while there were no significant differences in crude fat content between the 15% CP and 16% CP groups (*p* > 0.05). Additionally, different crude protein levels in the diet had no significant effect on the moisture content of Wanxi white goose eggs.

Furthermore, the content of proline (Pro) in the 16% CP group was significantly lower than that in the 14% CP group (*p* < 0.05), but no significant differences were observed between the 16% CP and 15% CP groups. In addition, the contents of Val, Met, and Tyr in 16% CP group were significantly decreased compared with the 15% CP group (*p* < 0.05).

## 4. Discussion

This study identified three main courtship behaviors of Wanxi white geese: male geese courting female geese, female geese courting male geese, and mutual courtship between males and females. The results showed that heterosexual interactions (gander to goose) accounted for the highest proportion (90.8–91.1%), followed by mutual courtship behaviors (5.3–6.4%), while homosexual interactions (gander to gander) represented the lowest proportion (2.7–3.6%). This suggests that males are dominant in the courtship process [23]. Research on Jinding ducks has indicated that they choose courtship partners based on vocalizations, feather color, and body shape [24], which is similar to the courtship behaviors observed in Wanxi white geese. This similarity suggests that the courtship performances of some waterfowl are generally comparable, although other types of waterfowl may exhibit different courtship methods. A previous study has reported that the breeding period of blue-eared pheasant begins in April, with few males consistently near females from mid-February [25]. Competing males will be aggressive toward each other in attempts to win mates through dueling. In contrast, the breeding season of Wanxi white geese likely extends from December to May of the following year, where multiple female geese tend to surround a single male. It is rare for several males to compete for a single female at the same time, differing from duel-based competition seen in blue-eared pheasants, which may be attributed to species differences. This study also found that the mating methods of Wanxi white geese primarily include normal mating and forced mating, consistent with the previous findings on east Zhejiang white goose [26]. Notably, it was observed that the primary mating method involves male geese climbing over female geese, and females may also forcibly seek out males during mating. Furthermore, the addition of 15% CP to the diet significantly increased the number of courtship and mating events in Wanxi white geese, thereby improving the fertilization rate of eggs. However, this dietary addition also significantly reduced the nesting frequency of Wanxi white geese. This study is the first to demonstrate that different levels of CP in diets can affect the reproductive behavior of Wanxi white geese, although the specific mechanism underlying these effects require further investigation.

Ovulation and egg production in poultry are regulated by various endocrine mechanisms, with hormone regulation playing a critical role in the reproductive process [27]. The synergistic or antagonistic effects of various reproductive hormones result in diverse and continuous reproductive activities. These hormones are essential for physiological processes such as follicular development, ovulation, and fertilization in poultry [28]. Estradiol and progesterone can synergistically promote egg production in female birds. The release of gonadotropin-releasing hormone from the hypothalamus stimulates the release of estradiol and progesterone, thereby improving the egg-laying performance of female birds. However, a previous study has indicated that high concentrations of progesterone can cause follicular atrophy and alter the nesting behaviors of female poultry [29]. It has been reported that serum estradiol levels increase with higher dietary protein levels [30]. In this study, while the addition of different CP levels in the diet did not significantly affect prolactin levels, the 16% CP group showed a significant increase in serum estradiol compared with the 14% and 15% CP groups [31]. FSH promotes the development and maturation of ovarian follicles and helps prevent atresia. It also enhances the reproduction of ovarian germ cells in chicken embryos, indicating that FSH is vital for the development of germ cells in poultry [32]. Luteinizing hormone (LH) stimulates follicular development and maturation, thereby promoting egg production [33]. During the laying period, poultry release FSH to continuously stimulates the production of follicles, with LH supporting this development [34]. Our results revealed that a diet containing 16% CP promotes the ovarian development of Wanxi white geese during the laying period, which is consistent with the observed concentrations of estrogen. A previous study has found that increasing dietary protein levels raised the concentrations of LH and progesterone in the blood of Zhedong white geese, while reducing the concentrations of FSH, estradiol, and prolactin during the laying period [35]. Our results showed that the concentrations of luteinizing hormone, progesterone, and GnRH in the 16% CP group were significantly higher than those in the 14% CP and 15% CP groups, indicating that a high protein diet could enhance the follicular development of Wanxi white geese during the laying period, consistent with the observed number of follicles. However, egg production in the 16% CP group did not significantly differ from that in the 14% and 15% CP groups. This discrepancy between follicular development and egg production may be related to the feeding environment [36].

Egg quality not only affects the edible value of eggs but also influences their market and commodity value. There are many factors affecting egg quality, including age, breed, and environmental conditions [37]. Our results indicated that the dietary addition of 15% CP can improve the nutritional quality of Wanxi white goose eggs. The specific gravity of an egg is a key indicator of its freshness—as eggs are stored longer, their specific gravity decreases. Fresh eggs typically have a specific gravity of more than 1.08, second-fresh eggs more than 1.06, stale eggs more than 1.05, and deteriorated eggs less than 1.05 [38]. Eggs with thin shells are more prone to breaking, while overly thick shells can hinder hatching and lead to stillbirths [39]. Eggshell color and egg yolk color are important criteria for consumers when judging egg quality. The b value of egg yolk color indicates the yellow-blue degree of the yolk; a larger b value corresponds to a lighter yolk color. This study found that different CP levels in the diet had no significant effect on egg yolk color. However, the eggshell yellowness in the 15% CP group was significantly higher than that in the 16% CP group. While altering the CP level in the diet did not significantly change the proportion of egg white to egg yolk, an increase in CP was associated with a gradual increase in the proportion of egg yolk.

Goose eggs have become a relatively common food, offering abundant protein and other essential nutrients. The protein in goose eggs includes all the essential amino acids required by the human body, known as complete proteins, which are highly absorbable. The yolk is particularly rich in phospholipids, which play a significant role in the development of the brain and nervous tissue. A previous study has shown that as protein levels in the diet increase, the nutritional value of the eggs improves, along with enhancements in flavor and palatability [40]. In a study measuring the crude fat, crude protein, and moisture content of Single Comb White Leghorn eggs, values were found to be 15.34%, 11.51%, and 73.35%, respectively [41]. This study detected 16 amino acids, with total amino acid contents varying under different crude protein levels at 43.18%, 43.72%, and 41.20%. Additionally, the crude fat and crude protein content in Wanxi White Goose eggs were found to be higher than those in Single Comb White Leghorn eggs, indicating that Wanxi White Goose eggs possess superior flavor and nutritional value.

## 5. Conclusions

In conclusion, this study is the first to identify a diet containing 15% crude protein (CP) as the optimal level for enhancing the reproductive performance, fertility, and egg specific gravity of Wanxi white geese during the laying period. By systematically analyzing the effects of dietary CP levels, we provide critical insights into the nutritional requirements of Wanxi white geese during this phase. These findings not only advance our understanding of their dietary needs but also lay a foundation for developing feeding standards that can improve management and productivity in this species.

## Figures and Tables

**Table 1:** Basal composition and nutrient levels (%) of experimental rations for laying Wanxi white geese, with different protein levels.

Item	Groups		
	14% CP	15% CP	16% CP
Diet composition (%)			
Corn	52.5	49.5	45.0
Soybean meal	17.5	20.5	23.0
Chaff mixing	8.0	8.0	8.0
Wheat bran	12.0	12.0	14.0
Stone powder	4.0	4.0	4.0
Premix for laying period of breeding geese ^1^	5.5	5.5	5.5
De-molding agent	0.5	0.5	0.5
Total	100.0	100.0	100.0
Nutrient level (%)			
Protein content	13.98	14.98	15.92
Crude fat	2.79	2.75	2.72
Ratio of energy to nitrogen	0.74	0.69	0.68
Crude fiber	5.47	5.53	5.67
Lysine	0.76	0.84	0.91
Methionine	0.26	0.27	0.28
Metabolizable energy (MJ/kg)	10.29	10.27	10.15

Note: ^1^ Each kilogram of basal diet contains: vitamin A, 9000 IU; vitamin D3, 3000 IU; vitamin E, 24 IU; vitamin K3, 1.8 mg; vitamin B12, 0.1 mg; riboflavin, 5 mg; nicotinic acid, 40 mg; pantothenic acid, 15 mg; pyridoxine, 3 mg; biotin, 0.05 mg; choline chloride, 500 mg; zinc, 90 mg; iron, 80 mg; manganese, 80 mg; copper, 20 mg; iodine, 0.35 mg. The feed composition in the table is based on the measured values.

**Table 2:** The kits used in this study.

Kits	Provider	No.
Total cholesterol assay kit	Nanjing Jiancheng Biotechnology Co., Ltd.	A111-1-1
The total protein assay kit		A045-3-2
Alkaline phosphatase assay kit		A059-1-1
Urea nitrogen assay kit		C013-1-1
Uric acid (UA) test kit		C012-2-1
Estradiol 2 assay kit		
Follicle stimulating hormone Assay kit		
Gonadotropin-releasing hormone		
Leptin assay kit		
Luteinizing hormone		
Progesterone assay kit		
Prolactin assay kit		

**Table 3:** Specific gravity of salt water in relation to salinity grade (0 to 8 g/cm^3^).

Grade	0	1	2	3	4	5	6	7	8
Specific gravity	1.068	1.072	1.076	1.080	1.084	1.088	1.092	1.096	1.100

**Table 4:** The reproductive behavior and performance of Wanxi white geese during the laying period (times per day).

Item	Crude Protein Levels			*p* Value
	14%	15%	16%	
Courtship frequency				
Male geese courting female geese	23.10 ± 0.65 ^ab^	23.43 ± 0.74 ^a^	22.25 ± 0.87 ^b^	0.032
Female geese courting male geese	0.93 ± 0.26	0.70 ± 0.21	0.88 ± 0.27	0.412
Mutual courtship between both sexes	1.43 ± 0.38	1.65 ± 0.29	1.30 ± 0.35	0.387
Female geese escape	4.00 ± 0.56	3.75 ± 0.36	3.75 ± 0.50	0.658
The frequency of mating				
Male geese climbing over female geese	31.20 ± 0.73 ^abc^	32.20 ± 1.14 ^a^	30.94 ± 0.77 ^b^	0.028
Female geese climbing over male geese	1.34 ± 0.34 ^ab^	1.46 ± 0.29 ^a^	1.08 ± 0.25 ^b^	0.047
Mating	28.32 ± 0.78 ^Bb^	29.96 ± 1.19 ^Aa^	28.32 ± 0.87 ^Bb^	0.003
Reproductive performance				
Nesting rate (%)	2.59 ± 0.59 ^ab^	2.19 ± 0.54 ^a^	3.16 ± 0.64 ^b^	0.039
Fertility (%)	73.19 ± 2.46 ^Bb^	84.35 ± 2.23 ^Aa^	71.37 ± 2.64 ^Bb^	0.001
Hatchability (%)	74.78 ± 2.65	73.67 ± 2.41	76.43 ± 3.02	0.521

Different capital letters indicate highly significant differences (*p* < 0.01), different lowercase letters indicate significant differences (*p* < 0.05), and the same letter indicates non-significant differences (*p* > 0.05) within the same line.

**Table 5:** The serum biochemical indices of Wanxi white geese during the laying period.

Index	Crude Protein Levels			*p* Value
	14%	15%	16%	
Total cholesterol (TC, mmol/L)	7.37 ± 0.75	4.75 ± 0.17	5.88 ± 0.30	0.127
Total protein (TP, mmol/L)	66.67 ± 1.91	60.00 ± 2.07	53.67 ± 3.23	0.089
Alanine aminotransferase (ALT, mmol/L)	10.33 ± 0.18 ^ab^	11.67 ± 0.48 ^a^	8.67 ± 0.48 ^b^	0.038
Alkaline phosphatase (ALP, mmol/L)	435 ± 13.32	413.33 ± 0.48	354.67 ± 21.40	0.156
Urea nitrogen (BUN, mmol/L)	0.47 ± 0.04	0.46 ± 0.02	0.43 ± 0.01	0.721
Uric acid (UA, mmol/L)	290.33 ± 10.51 ^b^	212 ± 12.32 ^a^	306.33 ± 11.87 ^b^	0.021
Calcium (CA, mmol/L)	3.98 ± 0.15	4.07 ± 0.13	3.51 ± 0.19	0.253

Different lowercase letters indicate significant differences (*p* < 0.05) and the same letter indicates non-significant differences (*p* > 0.05) within the same line.

**Table 6:** The concentrations of reproductive hormones of Wanxi white geese during the laying period.

Index	Crude Protein Levels			*p* Value
	14%	15%	16%	
Estradiol (E2, pg/mL)	13.83 ± 0.03 ^ABb^	9.04 ± 0.11 ^Aa^	15.86 ± 1.02 ^Bb^	0.003
Prolactin (PRL, mIU/mL)	200.10 ± 11.47	172.03 ± 8.85	215.14 ± 2.99	0.102
Follicle-stimulating hormone (FSH, mIU/mL)	11.92 ± 0.17	9.15 ± 0.26	14.56 ± 1.78	0.075
Luteinizing hormone (LH, mIU/mL)	5.46 ± 0.06 ^ABb^	4.25 ± 0.17 ^Aa^	6.18 ± 0.25 ^Bb^	0.002
Progesterone (P4, pg/mL)	27.78 ± 1.91 ^Bb^	16.46 ± 1.07 ^Aa^	34.59 ± 1.31 ^Bb^	0.001
Gonadotropin-releasing hormone (GnRH, pg/mL)	43.44 ± 1.88 ^Aa^	22.87 ± 0.66 ^Cc^	53.35 ± 1.18 ^Bb^	<0.0001
Leptin (LEP, mIU/mL)	6.80 ± 0.03 ^Bb^	7.40 ± 0.09 ^Aa^	6.21 ± 0.04 ^Cc^	<0.0001

Different capital letters indicate highly significant differences (*p* < 0.01), different lowercase letters indicate significant differences (*p* < 0.05), and the same letter indicates non-significant differences (*p* > 0.05) within the same line.

**Table 7:** The number of follicles of Wanxi white geese during the laying period.

Index	Crude Protein Levels			*p* Value
	14%	15%	16%	
Small white follicles	26.00 ± 0.63 ^ABb^	28.33 ± 1.28 ^Bb^	36.00 ± 0.84 ^Aa^	0.004
Large white follicles	4.67 ± 0.48 ^bc^	5.33 ± 0.48 ^b^	9.33 ± 0.80 ^a^	0.021
Small yellow follicles	3.33 ± 0.48	3.33 ± 0.18	5.00 ± 0.63	0.112
Large yellow follicles	2.00 ± 0.32 ^ab^	1.67 ± 0.36 ^b^	4.33 ± 0.48 ^a^	0.038

Different capital letters indicate highly significant differences (*p* < 0.01), different lowercase letters indicate significant differences (*p* < 0.05), and the same letter indicates non-significant differences (*p* > 0.05) within the same line.

**Table 8:** The laying performance of Wanxi white geese during the laying period.

Index	Crude Protein Levels			*p* Value
	14%	15%	16%	
Average egg weight (g)	160.2 ± 4.50	164.34 ± 5.90	160.30 ± 4.08	0.587
Daily egg production	5.70 ± 1.25	6.04 ± 1.11	6.10 ± 1.23	0.712
Feed-to-egg ratio	13.70 ± 4.60	11.54 ± 2.62	12.13 ± 3.24	0.453
Egg qualification rate (%)	96 ± 0.17	94.8 ± 0.40	96.3 ± 0.27	0.245
Average number of eggs laid by goose	18.7 ± 0.10	19.8 ± 0.07	20.0 ± 0.11	0.328

**Table 9:** The egg quality of Wanxi white geese.

Index	Crude Protein Level			*p* Value
	14%	15%	16%	
Egg index	1.45 ± 0.02	1.46 ± 0.03	1.44 ± 0.02	0.782
Eggshell thickness (mm)	0.58 ± 0.02	0.59 ± 0.02	0.57 ± 0.02	0.654
Albumen height (mm)	5.07 ± 0.28	5.49 ± 0.48	5.14 ± 0.35	0.413
Specific gravity of egg (g/cm^3^)	1.08 ± 0.01 ^ab^	1.09 ± 0.00 ^a^	1.07 ± 0.00 ^b^	0.038
Yolk color (b value)	42.70 ± 2.49	42.78 ± 2.27	43.99 ± 2.31	0.712
Yolk weight percentage (%)	37.47 ± 1.16	38.91 ± 2.62	38.57 ± 1.44	0.589
Albumen ratio (%)	48.34 ± 1.20	45.01 ± 3.47	46.32 ± 1.70	0.321
Eggshell color				
Brightness L	92.18 ± 0.79	92.05 ± 0.50	92.85 ± 0.52	0.423
Redness a	−1.20 ± 0.11	−1.28 ± 0.10	−1.39 ± 0.09	0.287
Yellowness b	7.77 ± 0.49 ^ab^	8.12 ± 0.50 ^a^	6.96 ± 0.52 ^b^	0.026

Different lowercase letters indicate significant differences (*p* < 0.05) and the same letter indicates non-significant differences (*p* > 0.05) within the same line.

**Table 10:** The nutrients and content of amino acid in Wanxi white geese eggs.

Index	Crude Protein Level			*p* Value
	14%	15%	16%	
Nutrients in eggs (%)				
Crude protein	47.91 ± 2.45	50.58 ± 1.26	47.27 ± 0.85	0.423
Crude fat	38.12 ± 0.46 ^b^	38.72 ± 0.35 ^ab^	40.00 ± 0.57 ^a^	0.031
Moisture	61.71 ± 0.51	59.85 ± 1.60	61.54 ± 0.68	0.287
The content of amino acid (%)				
Aspartate (Asp)	3.69± 0.36	4.14 ± 0.05	3.95 ± 0.05	0.356
Threonine (Thr)	2.70 ± 0.05	2.72 ± 0.03	2.58 ± 0.05	0.112
Serine (Ser)	3.56 ± 0.05	3.56 ± 0.04	3.41 ± 0.05	0.087
Glutamate (Glu)	6.26 ± 0.10	6.31 ± 0.07	5.36 ± 0.41	0.054
Proline (Pro)	1.59 ± 0.02 ^a^	1.55 ± 0.02 ^ab^	1.48 ± 0.02 ^b^	0.028
Glycine (Gly)	1.46 ± 0.02	1.47 ± 0.02	1.40 ± 0.03	0.098
Alanine (Ala)	2.12 ± 0.03	2.11 ± 0.03	2.04 ± 0.03	0.124
Valine (Val)	2.61 ± 0.04 ^ab^	2.63 ± 0.04 ^a^	2.47 ± 0.04 ^b^	0.037
Methionine (Met)	1.72 ± 0.04 ^ab^	1.77 ± 0.03 ^a^	1.61 ± 0.03 ^b^	0.042
Isoleucine (Ile)	2.00 ± 0.03	2.00 ± 0.03	1.94± 0.03	0.215
Leucine (Leu)	3.6 ± 0.05	3.58 ± 0.04	3.46 ± 0.04	0.087
Tyrosine (Tyr)	1.99 ± 0.03 ^ab^	2.00 ± 0.02 ^a^	1.90 ± 0.02 ^b^	0.039
Phenylalanine (Phe)	2.49 ± 0.05	2.51 ± 0.04	2.43 ± 0.10	0.654
Histidine (His)	2.22 ± 0.04	2.20 ± 0.03	2.08 ± 0.06	0.145
Lysine (Lys)	3.01 ± 0.02	3.05 ± 0.03	2.97 ± 0.03	0.187
Arg (Arg)	2.16 ± 0.03	2.11 ± 0.03	2.12 ± 0.03	0.432
Total amino acid	43.18	43.72	41.20	0.321

Different lowercase letters indicate significant differences (*p* < 0.05) and the same letter indicates non-significant differences (*p* > 0.05) within the same line.

## Data Availability

The original contributions presented in this study are included in this article and further inquiries can be directed to the corresponding authors.
